# Tracing the mobility of a Late Epigravettian (~ 13 ka) male infant from Grotte di Pradis (Northeastern Italian Prealps) at high-temporal resolution

**DOI:** 10.1038/s41598-022-12193-6

**Published:** 2022-05-16

**Authors:** Federico Lugli, Alessia Nava, Rita Sorrentino, Antonino Vazzana, Eugenio Bortolini, Gregorio Oxilia, Sara Silvestrini, Nicola Nannini, Luca Bondioli, Helen Fewlass, Sahra Talamo, Edouard Bard, Lucia Mancini, Wolfgang Müller, Matteo Romandini, Stefano Benazzi

**Affiliations:** 1 Department of Cultural Heritage, University of Bologna, Ravenna, Italy; 2 Department of Chemical and Geological Sciences, University of Modena and Reggio Emilia, Modena, Italy; 3 Human Osteology Lab, School of Anthropology and Conservation, University of Kent, Canterbury, UK; 4 Department of Biological, Geological and Environmental Sciences, University of Bologna, Bologna, Italy; 5 HUMANE - Human Ecology and Archaeology, Dept. Archaeology and Anthropology, Institució Milà i Fontanals de Investigación en Humanidades, Consejo Superior de Investigaciones Cientificas (IMF - CSIC), Barcelona, Spain; 6 MUSE, Museum of Science, Trento, Italy; 7 Departement of Humanities, Section of Prehistoric and Anthropological Sciences, University of Ferrara, Ferrara, Italy; 8 Department of Cultural Heritage, University of Padua, Padua, Italy; 9 Department of Human Evolution, Max Planck Institute for Evolutionary Anthropology, Leipzig, Germany; 10 Ancient Genomics Lab, The Francis Crick Institute, London, UK; 11 Department of Chemistry “Giacomo Ciamician”, University of Bologna, Bologna, Italy; 12 CEREGE, Aix Marseille Université, CNRS, IRD, INRAE, Collège de France, Technopôle de L‘Arbois, Aix-en-Provence, France; 13 Elettra - Sincrotrone Trieste S.C.P.A., Basovizza (Trieste), Italy; 14 LINXS – Lund Institute for advanced Neutron and X-ray Science, Lund, Sweden; 15 Frankfurt Isotope and Element Research Center (FIERCE), Goethe-Universität Frankfurt, Frankfurt am Main, Germany; 16 Institut Für Geowissenschaften, Goethe-Universität Frankfurt, Frankfurt am Main, Germany; 17 Pradis Cave Museum, Clauzetto, Italy

**Keywords:** Anthropology, Archaeology, Biogeochemistry

## Abstract

We present the results of a multi-disciplinary investigation on a deciduous human tooth (Pradis 1), recently recovered from the Epigravettian layers of the Grotte di Pradis archaeological site (Northeastern Italian Prealps). Pradis 1 is an exfoliated deciduous molar (Rdm_2_), lost during life by an 11–12-year-old child. A direct radiocarbon date provided an age of 13,088–12,897 cal BP (95% probability, IntCal20). Amelogenin peptides extracted from tooth enamel and analysed through LC–MS/MS indicate that Pradis 1 likely belonged to a male. Time-resolved ^87^Sr/^86^Sr analyses by laser ablation mass spectrometry (LA-MC-ICPMS), combined with dental histology, were able to resolve his movements during the first year of life (i.e. the enamel mineralization interval). Specifically, the Sr isotope ratio of the tooth enamel differs from the local baseline value, suggesting that the child likely spent his first year of life far from Grotte di Pradis. Sr isotopes are also suggestive of a cyclical/seasonal mobility pattern exploited by the Epigravettian human group. The exploitation of Grotte di Pradis on a seasonal, i.e. summer, basis is also indicated by the faunal spectra. Indeed, the nearly 100% occurrence of marmot remains in the entire archaeozoological collection indicates the use of Pradis as a specialized marmot hunting or butchering site. This work represents the first direct assessment of sub-annual movements observed in an Epigravettian hunter-gatherer group from Northern Italy.

## Introduction

Following the Late Glacial Maximum (LGM), glacial retreat and general climate amelioration from ~ 17,000 years ago (ka) allowed hunter-gatherer human groups to (re)expand into Northern Italy and Alpine areas^1^. From the Late Glacial to the onset of the Holocene, Italy was technologically characterized by the Epigravettian technocomplex, distributed across the whole peninsula and associated with a likely population replacement^2^. Indeed, genetic affinity with Near Eastern people from the Balkans characterizes the Villabruna cluster of Riparo Villabruna^3^ (ca. 14 ka) and Riparo Tagliente^1^ (ca. 17 ka).

The large number of well dated sites within the Alps across the Late Upper Paleolithic indicates a settlement network characterized by frequent logistic forays and movements, with possible contacts amongst hunter-gatherer human groups^4^. The high dynamics in terms of both population movements and climate fluctuations is also reflected in the subsistence strategies of the Epigravettian human groups^5^. Most of the previous work in Northern Italy consisted of archeozoological and archaeological analyses of settlements at several sites. Specifically, the links between variabilities in the lithic industry and site topographies indicate differential exploitation of valley floor camps and more specialized sites at higher altitudes^4^, with these latter mostly focused on specific hunting activities and frequented during summer-autumn months as attested by the faunal assemblages^6,7^. This is particularly evident at the onset of the Bølling–Allerød interstadial (ca. 14 ka), and the related expansion of (oak) forests when seasonal settlements moved to higher altitudes^5^.

Previous studies have utilized the strontium isotopic signature (^87^Sr/^86^Sr) of tooth enamel^8^ to investigate mobility patterns and strategies employed by human groups during the Pleistocene^9–14^. Specifically, the results by Lugli et al.^13^ showed a distinct change in mobility strategies for Epigravettian human groups from Paglicci (Southern Italy), compared with previous (i.e. Gravettian) hunter-gatherers. Another study on Early Upper Paleolithic Italian contexts investigated sub-annual movements of three Neanderthals and an Early Upper Paleolithic *Homo sapiens* using microchemical Sr-isotope data of deciduous teeth^12^. They found different mobility patterns between the Mousterian occupants who turned out to be mostly locals, and the *H. sapiens* who was probably non-local. Similarly, the lack of intra-tooth isotopic variability of a Middle Pleistocene human tooth from Southern Italy was interpreted to indicate limited mobility of the whole human group^11^. All the previously mentioned studies relied on deciduous human teeth, which additionally inform on the movements of the individual’s mother, due to their mineralization timespan which starts in-utero^15^. More specifically, combining histomorphometry on dental thin section of deciduous enamel and micro-chemistry, it is possible to reconstruct the mobility patterns and subsistence activities of pregnant women as well as of the child during early life (namely their ‘chemohistory’) at sub-monthly/weekly resolution^10,12,16–19^.

Yet, information concerning human mobility during the Upper Palaeolithic is still scarce and mainly inferred from indirect evidence such as raw materials^20^ and ornament sourcing^21^, hunting strategies and dietary choices^22^, and lower limb morphology^23^. Here we directly investigate the mobility and the life-history of an infant from the Epigravettian layers of the Pradis (or Grotte di Pradis) site^24,25^, through a multidisciplinary approach applied to an exfoliated deciduous tooth (sample ID: Pradis 1). Since Pradis 1 is unpublished, prior to histological sectioning and dentine sampling, a X-ray microtomographic record of the tooth was acquired to provide a three-dimensional (3D) morphological description and morphometric analysis of the human specimen. The tooth was then thin sectioned and analyzed using laser-ablation multi-collector inductively-coupled-plasma mass spectrometry (LA-MC-ICPMS) along the enamel-dentine junction, to collect ^87^Sr/^86^Sr data at a (sub)monthly resolution^26^. To better constrain the chronological context of the individual and, ultimately, to discuss mobility data within a detailed palaeoecological framework, a direct date was also obtained from dental collagen, exploiting a recently developed method for small specimens (< 100 mg bone/dentine material^27^). A small fragment of tooth enamel was employed for proteomic sexing through liquid chromatography mass spectrometry^28,29^, to decipher whether the tooth belonged to a male or a female.

Our results give direct insights into the seasonal mobility strategies of human groups during the Epigravettian, providing the only direct assessment for the Late Upper Paleolithic in Northern Italy and, ultimately, one of the few published to date. In addition, this deciduous tooth, lost *intra vitam* and thus informing on the biological life history of an individual who survived infancy*,* can provide direct first-hand information at high temporal resolution about the life of (pregnant) women and children within the social structure of a post-glacial hunter-gatherer group.

## Pradis archaeological site

**Figure 1:**
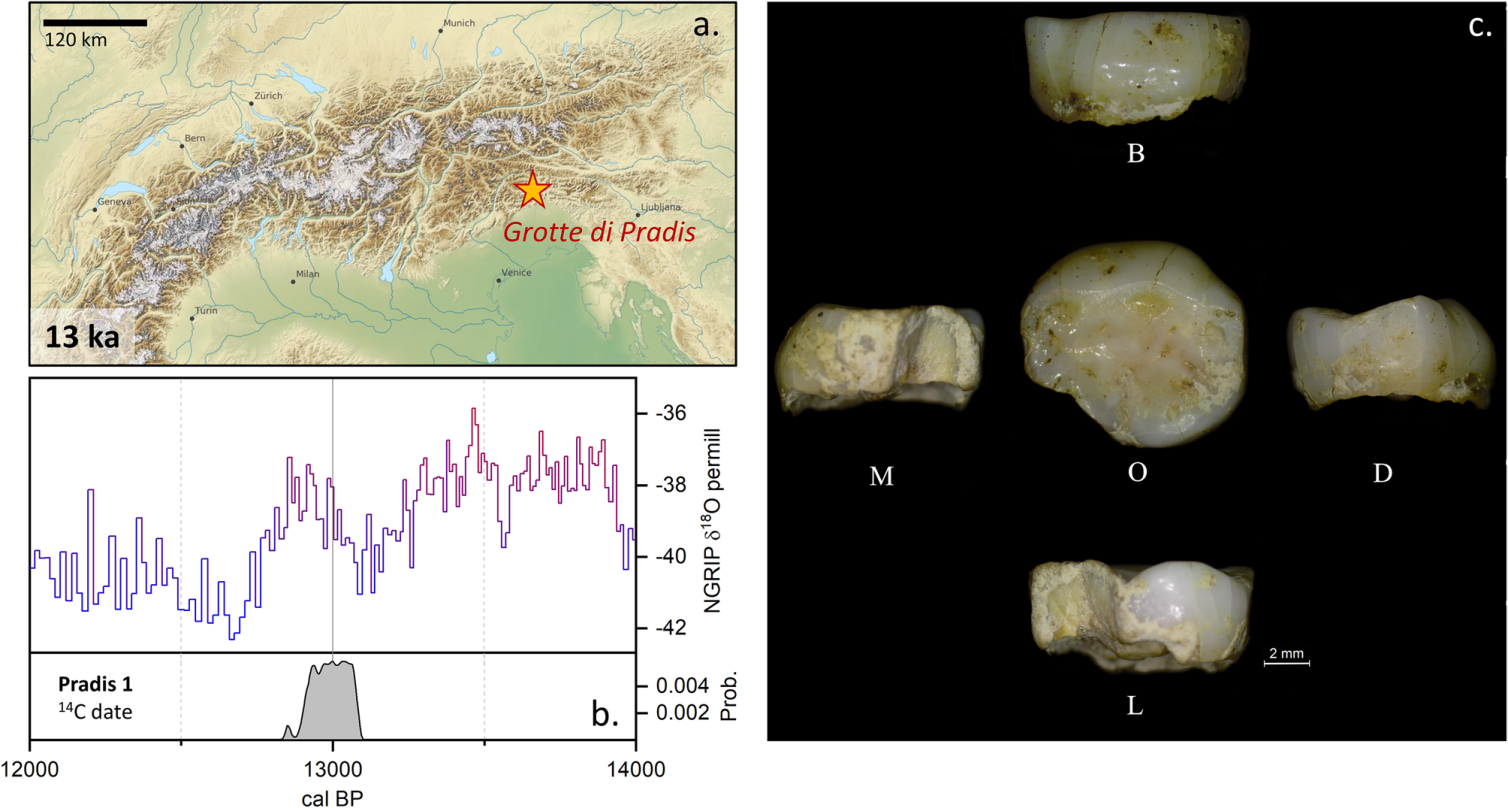
**(a)** Modeled Alpine glacier^83^ extent at 13 ka with the location of the Pradis site indicated by a star. **(b)** Direct radiocarbon dating of Pradis 1 likely placed this individual within the Bølling–Allerød interstadial (oxygen isotope data are from North Greenland Ice Core Project^84^). **(c)** Photographic record of Pradis 1 dm_2_ tooth; scale bar is 2 mm; *B* buccal, *D* distal, *L* lingual, *M* mesial, *O* occlusal.

The Pradis caves are located at 560 m a.s.l. on the Pradis Plateau (eastern side of the Carnic Prealps, NE Italy; Fig. 1). The orographic unit (ca. 850 km^2^) shows an irregular landscape, characterized by hills and valleys (400 to 800 m deep) and surrounded by mountains up to 2000–2300 m high.

The Pradis Plateau extends over an area of 6 km^2^, bordered by Mount Pala (1231 m), Mount Rossa (1369 m) and Mount Ciaurlec (1148 m), and by the gorge of the Cosa stream to the south. The plateau, located between the plain and the Prealps, plays a strategic role in accessing the mountain areas and the Tagliamento River upper basin. The hydrographic network exhibits several natural shelters and caves excavated in carbonate formations^25^. Pleistocene sediments filled some of these cavities, as Grotte Verdi di Pradis and Grotta del Clusantin, used during the Middle-Upper Palaeolithic.

The stratigraphic succession of Riparo I (i.e. the main shelter structure in the Pradis Caves) consists of a set of units mainly formed by the accumulation of cryoclastic gravel with variable content of silts and guano levels^24^. At the base, a thick layer of gravel with clayey matrix: levels 13 to 7, stripped by artificial levels, contained almost exclusively cave bear bones and some rare Mousterian lithic artefacts. Above, a level of guano (6) is covered with low silt gravel (5) and gravel with larger amount of silt (layers 4 to 2). Finally, layers 2 and 1 (the latter being split into 1a and 1b) yielded abundant bones of mammals, especially marmots, and the deciduous human tooth (Pradis 1) analysed in this paper^24^. Two radiocarbon dates on two cut-marked marmot bones (level 1a) indicate human frequentation between 13.9 and 12.6 ky cal BP^25^.

The lithic artefacts from levels 1 and 2 are homogeneous and belong to the Late Epigravettian; the flint is allochthonous, likely coming from the basin of the Venetian Prealps^30^. Along with lithic artefacts, bone tools have also been preserved: two bone points and two marmot clavicles with incised notches^24^ (layer 1a).

The associated fauna included large cervids (moose, red deer) and caprids (ibex and chamois) with cut and percussion marks, which testify the broad range of exploited herbivores by the Epigravettians^25^. However, the most targeted game was the alpine marmot (*Marmota marmota*), as evidenced by the fact that this species represents 99% of bone remains, i.e. number of identified specimens (NISP) > 11.000 and minimum number of individuals (MNI) = 637. Together with some projectile impact marks (PIMs) identified on marmot bones^31^ this huge bone assemblage testifies a capture strategy that avoided juvenile individuals, less appealing in terms of energetic return. Standardized butchering schemes suggest that the pelts were removed in a single piece, together with the paws and the tail and then transported out of the site, as for the meat recovered for a deferred consumption away from the site like a food storage. Unlike the neighbouring Clusantin^7^, data from the Pradis Caves testifies the building of a camp devoted to the intensive exploitation of a specific resource, within a well-established logistical mobility system at the end of the Upper Paleolithic^25^.

## Results

### Dental morphology

**Figure 2:**
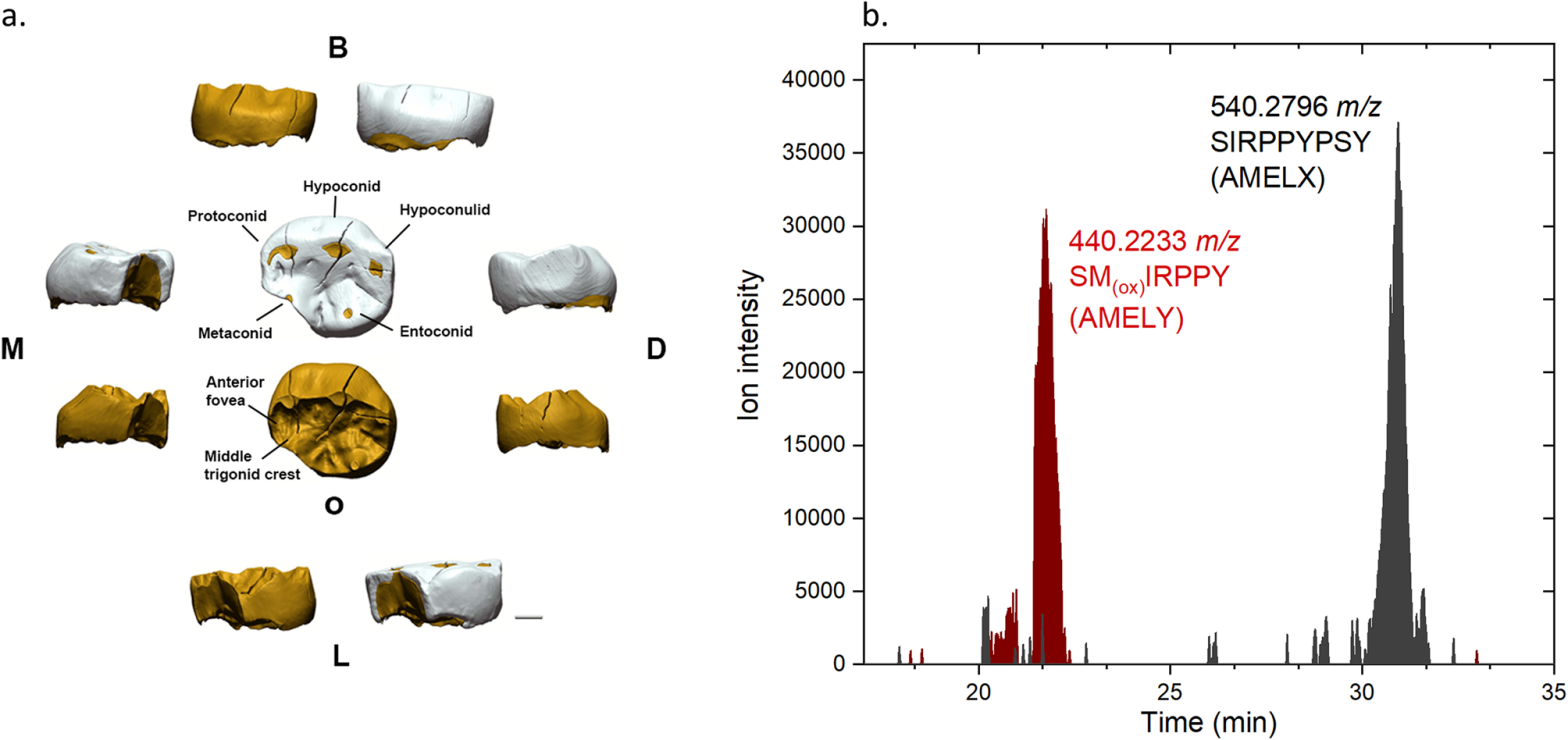
**(a)** Virtual reconstruction of Pradis 1 dm_2_ tooth showed through a 3D surface rendering, where white is enamel and dark yellow is the underlying dentine; scale bar is 2 mm; *B* buccal, *M* mesial, *L* lingual, *D* distal, *O* occlusal. **(b)** LC–MS ion chromatograms of peptides SM_(ox)_IRPPY and SIRPPYPSY^29^; the presence of SM_(ox)_IRPPY suggests that Pradis 1 was a male.

**Figure 3:**
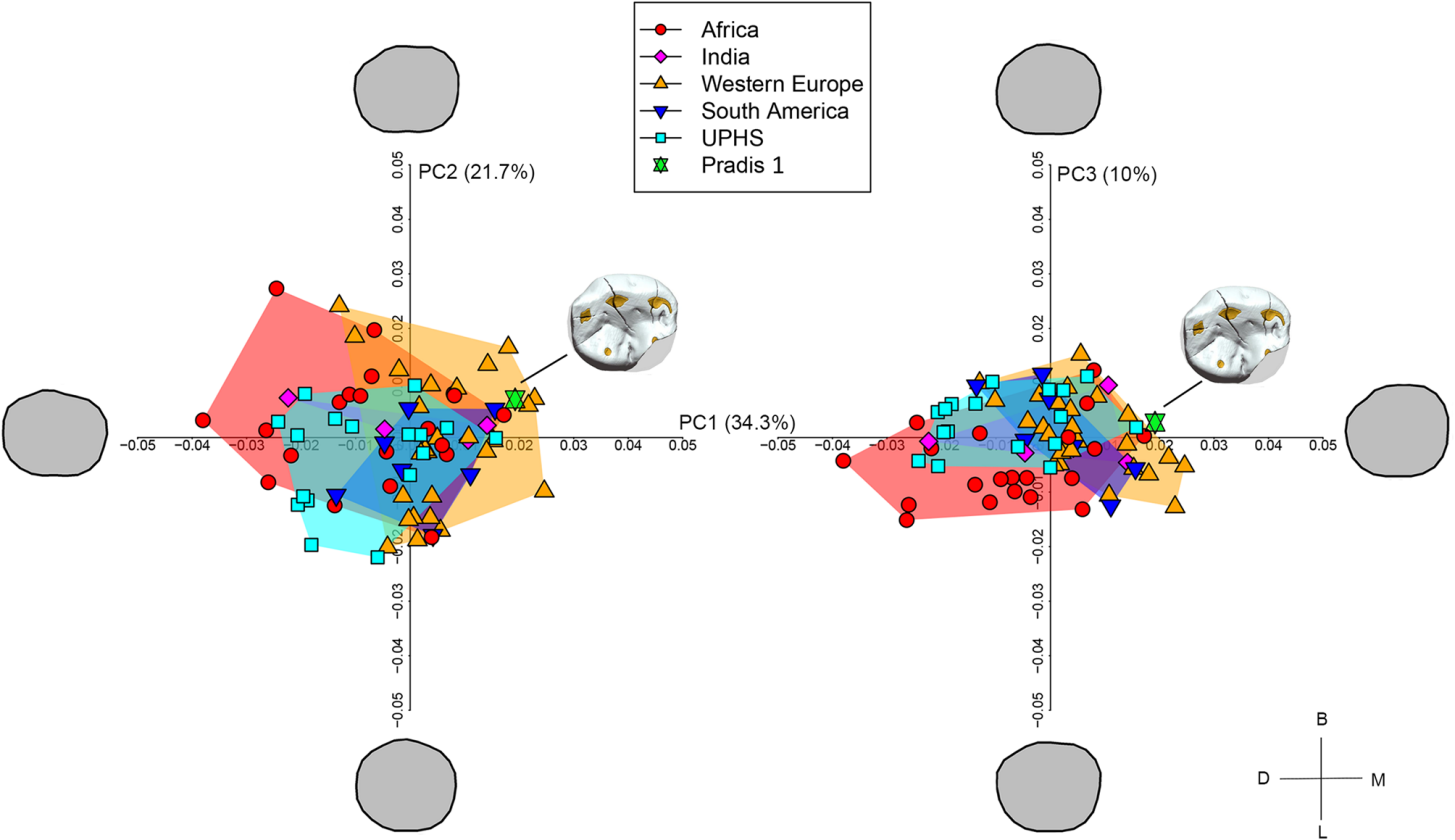
Principal component analyses (PCA) of left dm_2_crown outline of Upper Paleolithic (UPHS) and recent *H. sapiens* (Africa, India, Western Europe, and South America). The green star represents the Pradis 1 (right dm2 mirrored) reconstructed based on the pooled sample mean. The Pradis tooth is projected in the PCA plot (PC1 vs. PC2 on the left and PC1 vs. PC3 on the right).

Pradis 1 is an exfoliated lower right second deciduous molar (Rdm_2_), with the crown partially broken on its mesiolingual side and with three main fractures visible in the occlusal view that run from the buccal side to the central groove (Figs. 1c and 2a). The crown is moderately worn^32^ (wear stage 3) and on the distal wall an interproximal wear facet (length = 4.11 mm; height = 2.96 mm) attests the tooth was in contact with the first permanent molar. The interproximal mesial facet is also visible, albeit reduced in size by the mesiolingual fracture (length = 2.56 mm; height = 2.38 mm). On the occlusal surface, five main cusps, which create a Y fissure pattern (metaconid in contact with the hypoconid), and a weak anterior fovea can be recognized^33^. On the enamel-dentine junction (EDJ) a weak, but continuous, middle trigonid crest was observed^34^. The root is resorbed by more than three quarters (Res3/4 stage^35^), suggesting that the individual lost the tooth antemortem, at an age of about 11–12 years based on recent human standards^36^. After geometric morphometric reconstruction of the missing/worn dental crown portions (Suppl. Fig. S1), the crown outline of Pradis 1 was compared with a published dm_2_ sample of recent (RHS) and Upper Paleolithic *H. sapiens* (UPHS)^37^. Principal component analysis (PCA) plot shows a general overlap among UPHS and RHS groups (Fig. 3), although the Western European RHS mean significantly differs from the UPHS mean (p = 0.001) and the African RHS mean (p = 0.001) according to the permutation tests (N = 1000) based on the first three PCs (66% of variance). Pradis 1 plots in the area manly covered by the Western European RHS (i.e., positive scores of PC1) owing to an enlarged buccal outline, which reduces at the level of the hypoconulid, ultimately giving the tooth a more bucco-lingually bulging, less mesio-distally elongated, shape (Fig. 3).

### Proteomic sexing

A small fragment (< 1 mg) of enamel, a residue from histological sectioning, was digested with 1.2 M HCl and analysed by LC–MS/MS^29^. Although relatively low in terms of ion intensity (ca. 3–4 × 10^4^), the two peaks at 440.2233 m/z ([M+2 H]^+2^ peptide SM_(ox)_IRPPY) and 540.2796 m/z ([M+2 H]^+2^ peptide SIRPPYPSY) were present at the expected retention time (Fig. 2b; Suppl. Table S1), pertaining to AMELY and AMELX respectively^29,38^. In addition, peptide M_(ox)_IRPPY (AMELY) was detected at m/z 396.7073 (see Suppl. Fig. S2). Hence, Pradis 1 likely belonged to a child of male sex.

### Radiocarbon dating

The collagen yield, elemental and stable isotopic values of the Pradis 1 collagen extract were within the accepted ranges of well-preserved collagen^39,40^, indicating that it was suitably preserved for ^14^C dating (Suppl. Table S2). In particular, the tooth yielded 5.9% collagen (well above the ~ 1% minimum requirement), with a C:N ratio of 3.1. Likewise, the sample FTIR spectra was typical of well-preserved collagen with no evidence of exogenous material in the extract. The two radiocarbon measurements are statistically indistinguishable at the 95% confidence level (χ^2^ test: df = 1, T = 1.8 (5% 3.8), yielding a calibrated range of 13,088–12,897 cal BP (95% probability, IntCal20^41^; weighted mean using the R_Combine function in OxCal 4.4)^42^.

### Time-resolved Sr isotopic composition of dental enamel and enamel histology

**Figure 4:**
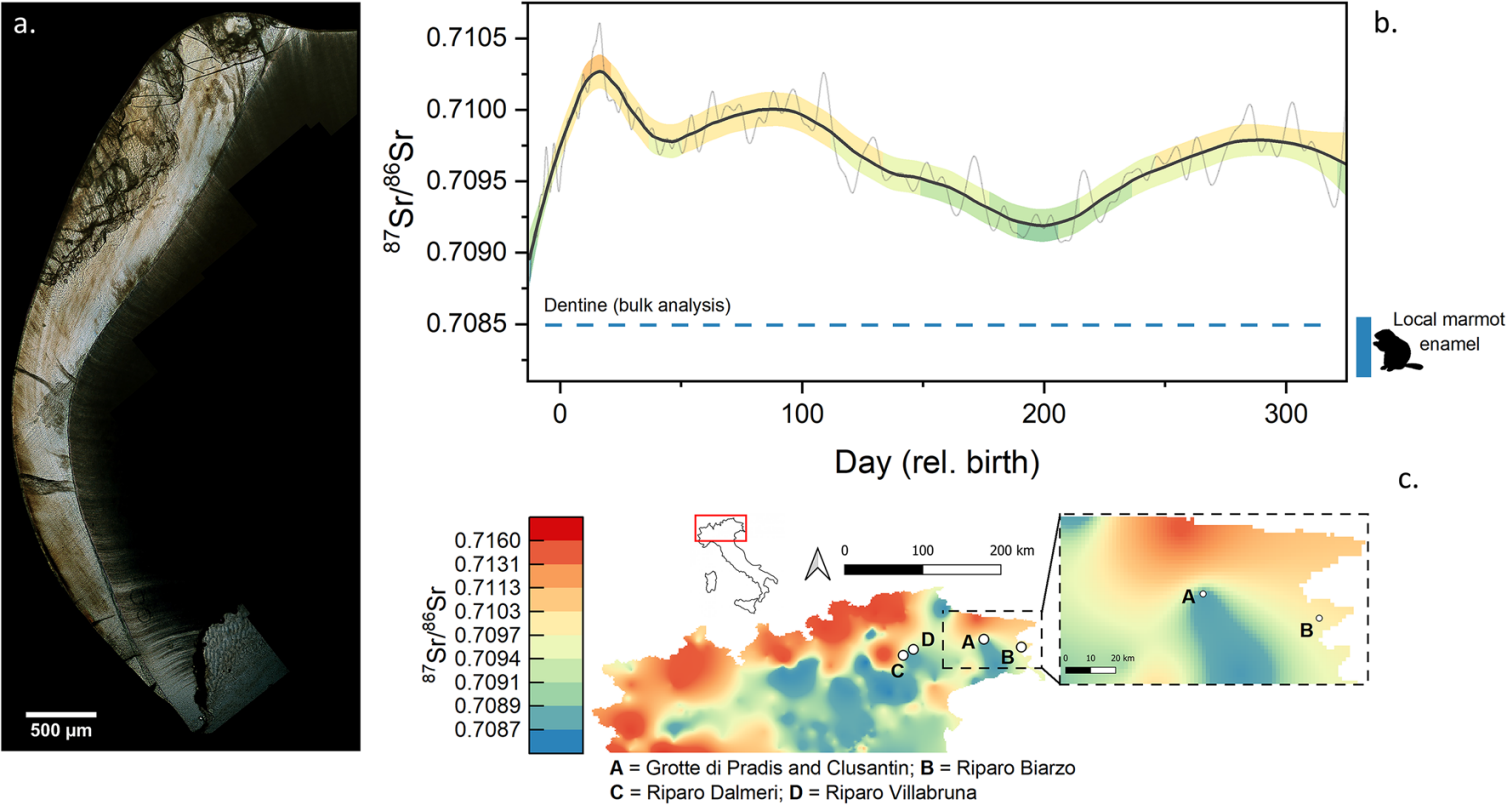
**(a)** Histological section of Pradis 1. **(b)** Time-resolved Sr isotope profile of Pradis 1 tooth enamel, smoothed through a locally weighted polynomial regression fit (LOWESS), with its associated standard error (± 2 SE). Note that the standard error bands are color-coded according to the isoscape ranges (see** c**); local marmot enamel and Pradis 1 dentine are reported as comparison. **(c)** Northern Italy isoscape based on Lugli et al.^52^; several relevant archaeological sites close to Pradis are reported.

The ^87^Sr/^86^Sr isotope data—obtained from histological thin section by LA-MC-ICPMS as a profile parallel to the EDJ (Fig. 4a,b)—ranges between 0.70880 and 0.71073, with clearly resolvable average minima and maxima of 0.70923 ± 9 (2 SE; 180–224 days) and 0.71023 ± 13 (2 SE; 9–26 days), respectively. The histological analysis of the dental crown allowed the chronologization of the profile registered through the neonatal line, i.e. the accentuated line that forms at birth^43^. This yielded ca. 338 days of the life of Pradis 1, spanning between ca. 13 days before and 325 days after birth. Any diagenetic alteration can be excluded for the enamel due to the U concentrations^12,44^, which for essentially the entire profile remained below the detection limit (~ 10 ppb; as determined through LA-ICPMS analyses).

### Definition of the local Sr isotope baseline

The local Sr isotope baseline was constrained using bulk Sr isotope analyses of archaeological marmot (*Marmota marmota*) enamel using MC-ICPMS^13^, yielding an average ^87^Sr/^86^Sr value of 0.70836 ± 0.00023 (2 SD, n = 5). Similarly, a bulk (ca. 1 mg) dentine ^87^Sr/^86^Sr value of 0.70850 ± 0.00001 (2 SE) was obtained for the Pradis 1 human tooth, which likely represents a mixture between the in-vivo signal and the local diagenetic end-member at Grotte Pradis. Both the Pradis 1 dentine and the marmot signals are consistent with the local geology. Pradis is surrounded (ca. 5 km in range) by Triassic dolomite and Cretaceous-Messinian limestone (Suppl. Fig. S3), whose isotope ratios range between 0.707 and 0.709 (from McArthur^45^). We acknowledge that the sole use of micromammal enamel, despite its early recommendation^46^, may underestimate the actual variability of the local bioavailable Sr pool. However, the use of ^87^Sr/^86^Sr ratios of modern plants can be biased by anthropogenic contaminants^47^ and/or glacial overprint. This is particularly true when modern environmental proxies are compared with archaeological specimens far back in time. Similarly, the use of snail shells is biased due to the high-contribution of soil carbonates to the snail diet^48–50^. For these reasons we cautiously chose to limit the comparison with archaeological tooth enamel of micromammals selected from the same stratum bearing the human tooth.

## Discussion

The specimen Pradis 1 is an exfoliated Rdm_2_ directly dated to 13,088–12,897 cal BP (95% probability), which was lost in life by an 11–12-year-old male child. Except for postmortem damage that affected the mesiolingual side of the crown, no dental pathologies or antemortem modifications (i.e., chippings, grooves) were present.

Morphologically, Pradis 1 presents characteristics that are common in *H. sapiens*, such as a straight lingual margin and a reduction of the distobuccal outline^37,51^. Moreover, the dm_2_ crown outline does not allow to clearly distinguish the UPHS from the RHS group as their range of variation overlaps (Fig. 3). Pradis 1 shares features mainly observed in Western Europe RHS, although not uniquely, such as an enlarged buccal outline that reduces distally, ultimately giving the crown a more rounded (i.e., less rectangular) shape.

The time-resolved ^87^Sr/^86^Sr signal recorded for Pradis 1 differs over the entire mineralization period of nearly one year (~ 340 days) from the Grotte Pradis site baseline, thus suggesting a non-local birthplace and residence area for this individual (Fig. 4b). Specifically, we can infer that his mother likely lived away from the site during the end of pregnancy and the first year of Pradis 1’s life. The high-resolution Sr isotope profile cyclically fluctuates from lower to higher Sr isotope values but never reaches the local end-member represented by Grotte Pradis baseline values (Fig. 4b). These fluctuations suggest that at least two different areas were visited by the Epigravettian human group—including infants—on an annual basis and likely following a seasonal mobility pattern. In fact, enamel histology facilitates the timing of the highest ^87^Sr/^86^Sr ratios of the tooth profile (> ~ 0.7097) to represent ~ 15 and ~ 100 days after birth and again between ~ 280 and ~ 325 days with lower values representing the ~ 3 intervening months. Such elevated ratios match several areas of the Northern Italian Alps^52^, whose bedrock geology for example include Variscan metamorphic rocks or various clastic rock formations, and overall suggest that the child was not born in Pradis.

The exploitation of the Pradis site on a seasonal, i.e. summer, basis is primarily indicated by the faunal spectra. Indeed, the nearly 100% occurrence of marmot remains in the entire archaeozoological collection indicates the use of Pradis as a specialized marmots’ hunting or butchering site. Given the marmot life cycle and based on data from the close Epigravettian site of Grotta del Clusantin^7^, this suggests that marmots were mostly hunted and consumed around September–October when their fat stores for hibernation peak at the highest levels.

**Figure 5:**
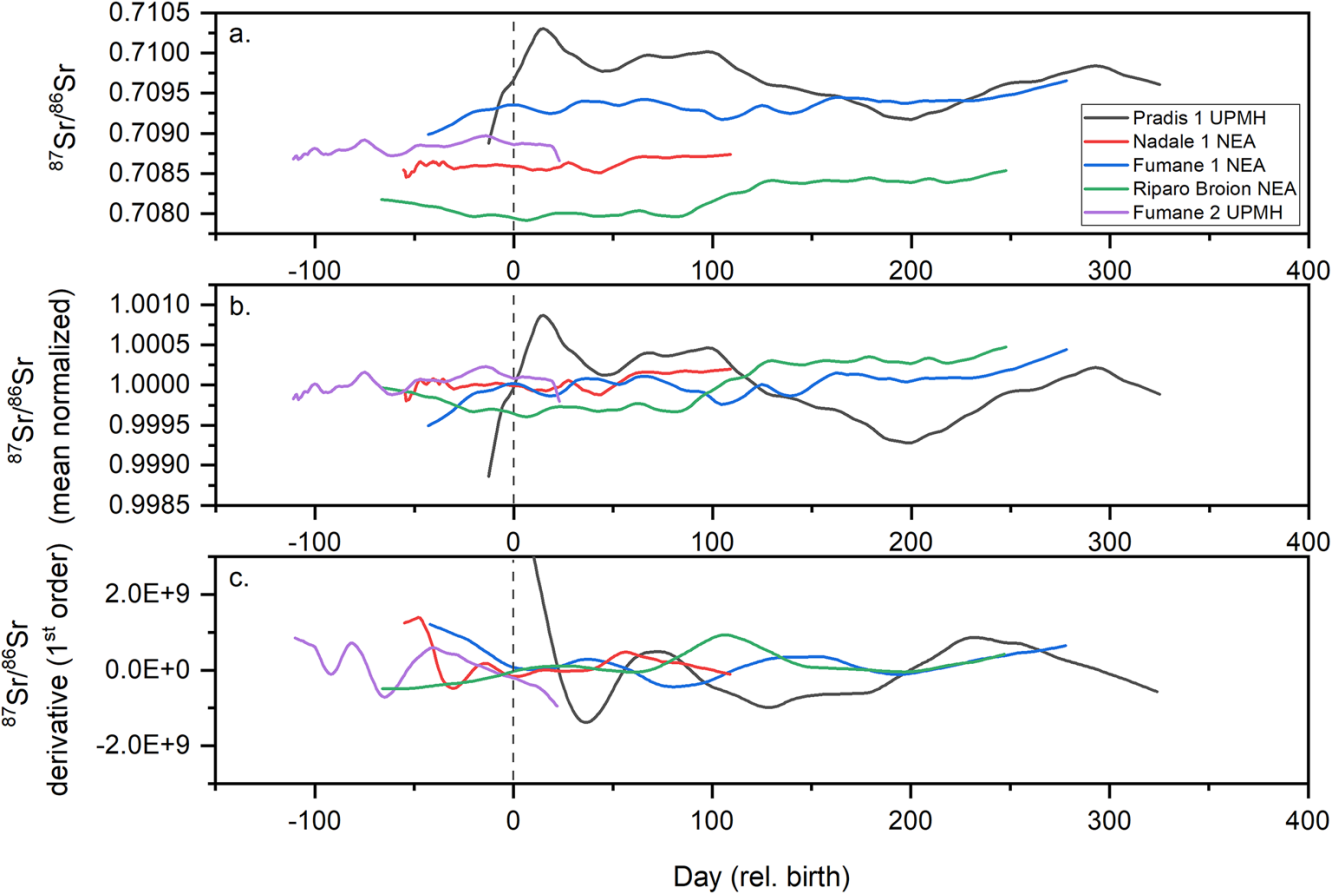
**(a)** Time-resolved Sr isotope profile of tooth enamel of Pradis 1 relative to respective results from other North-Italian Middle-Upper Palaeolithic *Homo* sp.^12^. **(b)** Same profiles as a. but normalized for their respective mean value. **(c)** First order derivative of the ^87^Sr/^86^Sr profiles from (**a**). *NEA* Neanderthal, *UPMH* upper palaeolithic modern human.

It is worth noting that variations in Sr content and isotope compositions of the different reservoirs, physiology and selective feeding behaviors may complicate the interpretation of high-resolution Sr isotopic profiles, as recently suggested for caprids^53^. It is also noteworthy that along the tooth growth, the ^87^Sr/^86^Sr profile shows changes in the slope of the variation, as shown by the first derivative of the mean normalized data (Fig. 5). Deriving such data helps visualizing and quantifying the variability along time-series profiles and evaluating possible mobility patterns^53^. In particular, the largest changes in the first derivative are evident pre-natally and during the first weeks after birth for Pradis 1 (but see also Fumane 2 and Nadale 1 from Nava et al.^12^, Fig. 5). The slope variations can be influenced by several environmental/dietary factors (i.e. food end-members with different Sr contents).

Altogether this evidence suggests that Pradis 1 did not dwell at the site for a long time period during the first year of his life, if at all. However, assuming for example that body Sr did not have enough time to reach the low radiogenic end-member (i.e. Pradis, ~ 0.7085) due to a slow turn-over, we can alternatively speculate that the lowering (0.7088–0.7092) of the ^87^Sr/^86^Sr signal in the tooth profile could have resulted from visiting Grotte Pradis or from the heavy reliance of food gathered at Grotte Pradis (i.e. stored marmot meat)^25^.

The direct radiocarbon dating of Pradis 1 placed this individual within the Bølling–Allerød interstadial, a period witnessing dense reforestation^54^ of the Alpine area after the LGM peak (Fig. 1). This evidence seems to support the likely seasonal mobility pattern we observed for the Epigravettian from Pradis. In temperate forests, indeed, mobility tends to be constrained by seasons, advocating the exploitation of patchily distributed resources by targeted forays^55^. Compared with other data from Northern Italy concerning different periods and hominin taxa (i.e. Neanderthal and *H. sapiens*), Pradis 1 shows the largest intra-tooth variability reported so far (Δ_max-min_ =  ~ 0.0015).

**Figure 6:**
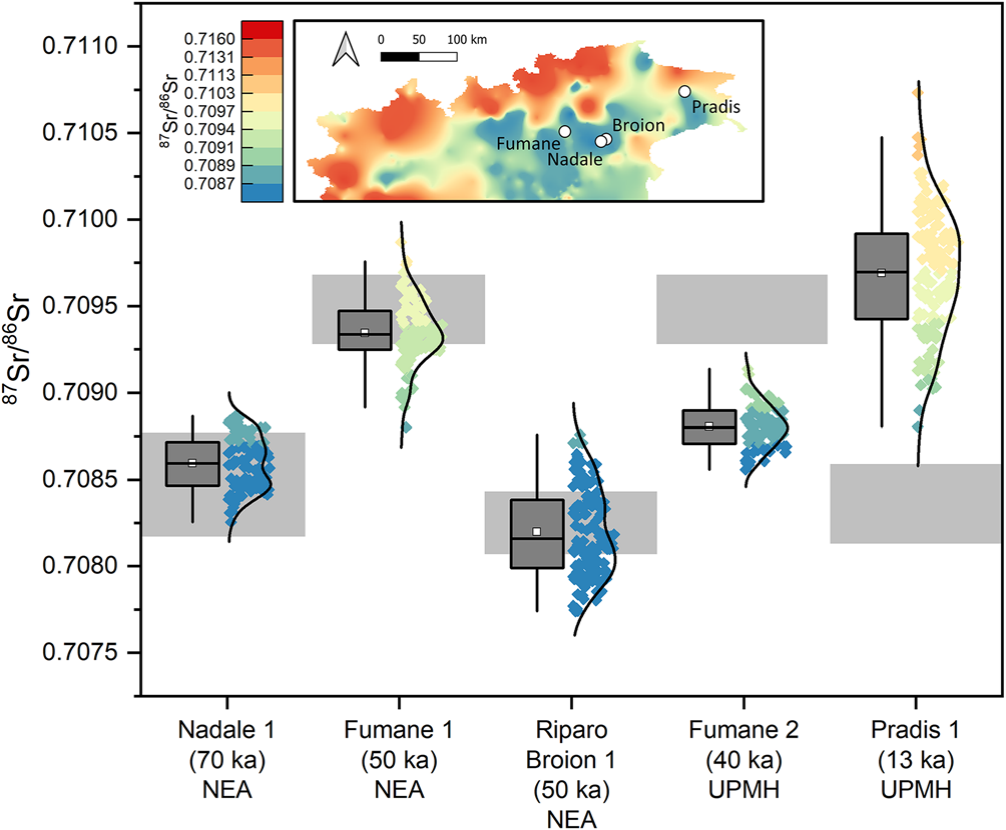
Box plot representing intra-tooth ^87^Sr/^86^Sr data of Northern Italy Neanderthals and UP humans from this study and from Nava et al.^12^. Data distribution is also reported and color-coded following the palette of the isoscape in the inset. Grey areas are local baselines, defined through micromammal tooth enamel. *NEA* neanderthal, *UPMH* upper palaeolithic modern human.

The ^87^Sr/^86^Sr values of earlier Neanderthal teeth on average overlap with their respective local baselines, possibly suggesting a limited mobility pattern and also a relatively narrow home range^12^ (see Fig. 6). In contrast, the two UPMHs (Fumane 2 and Pradis 1) are not compatible with the local bioavailable Sr indicated through the micromammals (Fig. 6), hinting at a larger home range of the *H. sapiens* groups and/or a different mobility strategy compared to Neanderthals^12^. Such evidence challenges observations in previous studies from other geographical areas. For example, Wißing et al.^56^ analysed UPMHs and Neanderthals from Spy and Goyet in Belgium. Their data suggest similar diets but different mobility strategies for the two human taxa. Specifically, Goyet Neanderthals were possibly exploiting non-local food resources based on their δ^34^S collagen fingerprint. On the other hand, Goyet UPMHs and Spy Neanderthals relied on local foodstuffs. In general, Neanderthals (with the exception of those analysed from Italy so far) show non-local^9^ (but see^57^) or semi-local^14^ Sr isotope values in their tooth enamel, supporting the idea of relatively large home ranges. Unfortunately, Sr isotope data for UPMH are scarce, which limits our possibility for direct comparison. At Grotta Paglicci, Lugli et al.^13^ observed different mobility patterns for Gravettians and Epigravettians, with the latter displaying non-local Sr isotope values but narrower intra-tooth variability. Altogether, our current knowledge does not allow to infer general behavioral models distinguishing Neanderthals from *H. sapiens*. Although climate and environmental fluctuations can be possible driving factors for large scale movements, the local distribution of resources might be a more parsimonious explanations for the mobility patterns observed, with the additional contribution of bio-cultural adaptation strategies exploited by different human groups.

Pradis 1 adds a further piece to disentangle the puzzle of human mobility during the Paleolithic period. This ca. 11–12 years old male child likely spent his first year of life moving across the Northeastern Italian landscape with his mother or, in general, the whole human group, during the Bølling–Allerød climatic amelioration (ca. 13 ka). Such evidence reinforces our (scant) knowledge on Late Epigravettian mobility patterns, providing this first direct assessment of seasonal movements for these hunter-gatherer human groups.

## Methods

### Dental morphology

The morphological description of the tooth is based on a terminology reported in dental anthropological literature^33^, whereas occlusal wear stages are scored based on Molnar^32^. Age at death (or alternatively the assessment of tooth loss ante-mortem) was estimated by combining the evaluation of tooth formation^36^ and root resorption^35^.

The X-ray microtomographic analysis of Pradis 1 was carried out by using the TomoLab station at the Elettra synchrotron facility (Basovizza, Trieste, Italy)^58^ and a virtual 3D reconstruction of the tooth was obtained using an isotropic voxel size of 5.5 μm. The TomoLab station is based on a sealed microfocus source (Hamamatsu L9181, minimum focal spot size: 5 μm). A set of 2400 projections over a 360° total scan angle was acquired using a water-cooled, 12bit, 4008 × 2672 CCD camera (VHR, Photonic Science) with an effective pixel size of 12.5 μm. The scan was carried out at a Voltage of 130 kV, a current of 61 μA and an exposure time/projection of 7.0 s applying a binning = 2 × 2 to the detector pixels. The slice reconstruction was done by the commercial software COBRA (Exxim, USA). The same software was used for beam hardening artifacts correction. The Pore3D software^59^ was applied to the reconstructed axial slice for ring artifacts removal.

The µCT image data were semiautomatically segmented using Avizo 9.2 software (Thermo Fisher Scientific, Waltham, Massachusetts, US), and the 3D models of the dental tissues (i.e., enamel and dentine) were refined in Geomagic Design X (3D Systems Software, Rock Hill, South Carolina, US) to optimize the triangles and create fully closed surfaces (Fig. 2a).

The 3D model of Pradis 1 was used for the morphological description and analyses of the tooth aided by virtual methods^51,60^, as well as for the restoration finalized to preserve the original state of the tooth before micro invasive analyses (see Suppl. Fig. S4).

The crown outline of Pradis 1 was compared with the Upper Paleolithic and recent *H. sapiens* (UPHS and RHS, respectively) left dm_2_ sample published in Bailey et al. following the procedure published in several works^37,51,61^. Briefly, the 3D model of the tooth was oriented with the cervical border perpendicular to the optical axis in both mesio-distal and bucco-lingual directions in Avizo 9.2 (Thermo Fisher Scientific), and a screenshot of the oriented tooth was saved in a jpeg file^62^. The missing portions of the tooth (distal interproximal wear and mesiolingual fracture) were firstly manually reconstructed in Adobe Photoshop®^37,51,63,64^ (Suppl. Fig. S1a), then the Pradis 1 right dm_2_ was mirrored in order to be analyzable with the left dm_2_ comparative sample. The jpeg file with the tooth mirrored and restored was imported in the Rhino 4.0 Beta CAD environment (Robert McNeel & Associates, Seattle, WA). Here, the tooth was oriented with the lingual side parallel to the x-axis^51^ and the crown outline was manually digitized using the spline function. The outline was centered superimposing the centroids of its area and the outline subdivided by 24 equiangularly spaced radial vectors emanating from the centroid (the first radius parallel to the y-axis and buccally directed). This results in the formation of 24 pseudolandmarks that were scaled to unit centroid size becoming Procrustes shape coordinates that were ultimately used to explore shape variation through principal component analysis (PCA; e.g.^37,51,61,65^). Since geometric morphometrics offers the possibility to objectively estimate missing landmarks^66,67^, we used the function “estimate.missing” in the R package Geomorph v3.3.1^68^ based on thin-plate spline interpolation (TPS) to estimate the pseudolandmarks falling in the mesiolingual fracture (n = 5) and distal interproximal area (n = 2) of Pradis 1 by using as a reference the mean of the UPHS and African, Indian, Western European, South American, and RHS-UPHS pooled groups, respectively (Suppl. Fig. S1a). The 6 reconstructed crown outlines of Pradis 1, together with that one manually reconstructed, were projected in the shape space PCA of the UPHS and RHS sample in order to assess if the reference mean may affect the final outcome (Suppl. Fig. S1b). Since the reference choice did not affect the results (Suppl. Fig. S1b), we used the Pradis 1 reconstructed based on the overall sample mean (i.e., RHS-UPHS pooled groups) to be projected in the shape space PCA of the comparative sample. Finally, permutation tests (n = 1000) using the first three PCs were conducted to identify potentially significant differences in crown shape among UPHS and RHS groups using the R package Morpho v. 2.8^69^.

### Physical restoration after sampling

The applied protocol for physical restoration involves various stages of work^70^, namely: (a) X-ray microtomographic acquisition and digital reconstruction of Pradis 1 in Geomagic Design X (as detailed above); (b) sampling of the specimen (see above); (c) microtomographic acquisition and digital reconstruction of Pradis 1 after sampling in Geomagic Design X; (d) superimposition of the two digital models (i.e., before and after sampling) using the iterative closest point method in Geomagic Design X and creation of a best-fit plane on the cut surface of the fragment removed during the sampling, which was used to digitally identify and isolate the sampled portion from the original digital model of Pradis 1; (e) creation of an exact replica of the sampled portion by rapid prototyping technology with an Orange 10 LCD 3D printer, using Longer UV resin, layer thickness 0.05 mm, UV Matrix 405 nm LED lighting sources and slicing software Longerware; (f) painting of the replica and restoration the original specimen Pradis 1 by using compatible and reversible adhesives. A post-restoration photographic record of the tooth is reported in Suppl. Fig. S4.

### Proteomic sexing

Owing to the dimorphic features of specific amelogenin isoforms, proteomics is currently revolutionizing the way of sexing ancient teeth^28^. A small fragment (< 1 mg) of enamel, left from histological sectioning, was washed in an ultrasonic bath with MilliQ water and digested through 1.2 M HCl (Suprapur). The dissolved specimen was purified and desalted through HyperSep SpinTips (Thermo Scientific) with C18 functionalized silica, following the protocol described in Lugli et al.^29,38^. Subsequent LC–MS analyses were performed at the Centro Interdipartimentale Grandi Strumenti of the University of Modena and Reggio Emilia by means of a Thermo Scientific Dionex Ultimate 3000 UHPLC coupled to a Thermo high-resolution Q Exactive mass spectrometer (Thermo Scientific, Bremen, Germany). The resulting ion chromatogram was manually inspected using Xcalibur (Thermo Scientific), looking for specific amelogenin peptides^29^. The ICIS algorithm ox Xcalibur was employed for automatic peak detection. Mascot searches were performed against SwissProt (constrained to *Homo sapiens*) and cRAP for contaminants. No proteolytic enzyme was selected, deamidated asparagines/glutamine (NQ), oxidated methionine (M) and phosphorylation (ST) were set as variable modifications in the search parameters. Mass tolerances were set at 10 ppm for the precursor ions (peak detection mismatch #^13^C = 1) and 0.05 Da for the product ions. An automatic decoy database search was used to estimate the false discovery rate (< 1%). Only amelogenin (razor AMELX) and no contaminants have been identified in the extract. The mass spectrometry proteomics data have been deposited to the ProteomeXchange Consortium via the PRIDE^71^ partner repository with the dataset identifier PXD030546.

### Radiocarbon dating

Collagen was extracted from the Pradis 1 tooth following the pretreatment protocol described in Fewlass et al.^27^ for bone/tooth samples < 100 mg, including demineralization, NaOH treatment and ultrafiltration.

To assess the quality of the collagen extract based on elemental and isotopic data^39,40^, ~ 0.5 mg collagen was weighed into a tin cup and analyzed with a Thermo Finnigan Flash elemental analyzer (EA) coupled to a Thermo Delta plus XP isotope ratio mass spectrometer to obtain elemental (C%, N%, C:N) and stable isotopic data (δ^13^C, δ^15^N). Carbon and nitrogen stable isotope values were two-point scale normalized to the VPDB (Vienna PeeDee Belemnite) and AIR (atmospheric N_2_) scale respectively using IAEA-CH-6 (sucrose, δ^13^C = − 10.449 ± 0.033 ‰), IAEA-CH-7 (polyethylene, δ ^13^C = − 32.151 ± 0.050 ‰), IAEA-N-1 (ammonium sulfate, δ^15^N = 0.4 ± 0.2 ‰) and IAEA-N-2 (ammonium sulfate, δ^15^N = 20.3 ± 0.2 ‰). An in-house methionine standard was used a quality control which gave average values of δ^13^C = − 28.23 ± 0.06 ‰ (1 S.D.) and δ^15^N = − 5.59 ± 0.08 ‰ (1 S.D). As a further quality check due to the small sample size, a small aliquot of collagen (~ 0.3 mg) was homogenized and mixed with ~ 40 mg of IR grade KBr powder in an agate mortar and pestle and pressed into a pellet using a manual hydraulic press (Wasserman) for analysis with an Agilent Technologies Cary FTIR Spectrometer with a DTGS detector^72–75^. Spectra were recorded in transmission mode at 4 cm^−1^ resolution with averaging of 34 scans between 4000 and 400 cm^−1^ using Resolution Pro software (Agilent Technologies).

The extracted collagen was radiocarbon dated using two accelerator mass spectrometer (AMS) measurement techniques to cross-check the results. Firstly, ~ 3.5 mg collagen was weighed into a pre-cleaned tin cup and sent to the Curt-Engelhorn-Centre for Archaeometry Klaus-Tschira-AMS facility in Mannheim, Germany (lab code: MAMS) for graphitization and dating with the MICADAS-AMS^76^. Secondly, a small extract of collagen (~ 0.24 mg) was dated using the gas ion source of the AixMICADAS^77^ at CEREGE (Centre de Recherche et d’Enseignement de Geosciences de l’Environnement, Aix-en-Provence, France; lab code: AIX), through the protocol described in Tuna et al.^78^ and Fewlass et al.^27^.

### Time-resolved Sr isotope analyses by LA-MC-ICPMS

To obtain ^87^Sr/^86^Sr data at high time resolution, Pradis 1 was first thin sectioned following standard histological protocols as described in Nava et al.^12,79^. The thin section of the dental crown was prepared at the Service of Bioarchaeology of the Museo delle Civiltà in Rome. The sectioning protocol consists of a detailed embedding-cutting-mounting procedure that makes use of dental adhesives, composite resins, and embedding resins. The tooth was first covered with a thin layer of reversible resin (Crystalbond 509, SPI Supplies) that does not contaminate chemically the dental tissues and which is soluble in Crystalbond cleaning agent (Aramco Products, Inc.). This step allows the removal of the crown from the resin block after thin sectioning to perform the restoration. The second embedding in epoxy resin (EpoThin 2, Buehler Ltd), cured for 24 h at room temperature, guarantees the protection of the sample during the cutting procedure. The tooth was sectioned using an IsoMet low-speed diamond blade microtome (Buehler Ltd). After the first cut, a microscope slide was attached to the exposed surface using the epoxy resin. A single longitudinal bucco-lingual thin section, averaging 250 µm thick, was cut from the specimen. The thin section was ground using water resistant abrasive paper of different grits (Carbimet, Buehler Ltd) to a final thickness of ~ 150 µm and polished with a micro-tissue (Buehler Ltd) and diamond paste with 1 µm size (DB-Suspension, M, Struers).

The thin section was imaged with a transmitted light microscope (Olympus BX 60) under polarized light, with different magnifications (× 40, × 100, × 400). Overlapping pictures of the dental crown captured through a digital camera (Nikon DSFI3) were assembled in a single photomosaic using the software ICE 2.0 (Image Composite Editor, Microsoft Research Computational Photography Group).

LA-MC-ICPMS analyses were performed at the Frankfurt Isotope and Element Research Center (FIERCE) at Goethe University, Frankfurt am Main (Germany) on the histological thin section, following the methodology detailed in^26^. Acquisition was performed in slow continuous profiling mode in enamel closest to (and < 100 μm from) the enamel-dentine junction, in the direction of tooth growth^12,19,26^. A 193 nm ArF excimer laser (RESOlution S-155, formerly Resonetics, ASI, now Applied Spectra Inc.) equipped with a two-volume LA cell (Laurin Technic) was connected to a NeptunePlus (ThermoFisher) MC-ICPMS using nylon6-tubing and a ‘squid’ signal-smoothing device^80^. Ablation took place in a He atmosphere (300 ml/min), with ~ 1000 ml/min Ar added at the funnel of the two-volume LA cell and 3.5 ml/min N_2_ before the squid. Laser fluence on target was ~ 5 J/cm^2^. Tuning of the LA-MC-ICPMS used NIST 616 glass for best sensitivity (^88^Sr) while maintaining robust plasma conditions, i.e. ^232^Th^16^O/^232^Th < 0.5% and ^232^Th/^238^U > 0.95 with RF-power of ~ 1360 W. In view of the low enamel [Sr] between 50 and 75 µg/g, we utilized 130 μm spots, a scan speed of 5 μm/s and a repetition rate of 20 Hz to obtain ^88^Sr ion currents of 1.6–2.6 × 10^–11^ A. Nine Faraday detectors were used to collect the ion currents of the following masses (m/z): ^83^Kr, ~ 83.5, ^84^Sr, ^85^Rb, ^86^Sr, ~ 86.5, ^87^Sr, ^88^Sr, ^90^Zr. The isotopically-homogenous (Sr) enameloid of a modern shark was repeatedly used to assess accuracy of the Sr-isotopic analysis and yielded ^87^Sr/^86^Sr = 0.70917 ± 2 and ^84^Sr/^86^Sr = 0.05652 ± 5 (2 S.D.; n = 5). Fully-corrected Pradis 1 ^87^Sr/^86^Sr, ^84^Sr/^86^Sr and ^85^Rb/^86^Sr ratios are listed in Supplementary Dataset S1. Any minor residual variability in ^84^Sr/^86^Sr is likely due to Kr-background variations not fully accounted for by the extended on-peak baseline measurement, in view of elevated Kr-contamination of the plasma-support gas Ar on the day of analysis.

The chronologies (of enamel secretion time) in days/months of life along the laser tracks were obtained by matching the linear profiles with the enamel microstructural incremental features^12^.

To better constrain human mobility (Fig. 4), an isoscape of Northern Italy was built using literature data (https://www.geochem.unimore.it/sr-isoscape-of-italy/) of bioavailable Sr^52^. Specifically, geolocated ^87^Sr/^86^Sr data of plants, waters, soils (bioavailable fraction) and biominerals have been collected (n = 883) and plotted using QGIS (version 3.18). Ordinary kriging was employed to interpolate missing ^87^Sr/^86^Sr data, best fitting the semivariogram through a linear model (SAGA 7.9)^52^.

First order derivatives of the Sr isotope profiles over time (Fig. 5) were obtained using R, with a lag parameter = 1. Derivatives were calculated after resampling the ^87^Sr/^86^Sr profiles at one-day resolution.

### Solution Sr isotope analyses

Sr baseline samples were processed at the Geochemistry Lab of the Department of Chemical and Geological Sciences (University of Modena and Reggio Emilia). A small dentine fragment from Pradis 1 (ca. 1 mg) was cleaned with MilliQ water in a sonicator, leached in ~ 0.5 M HNO_3_ and then digested in concentrated HNO_3_. Enamel fragments of marmots’ teeth were manually separated from the dentine using a scalpel. These fragments were then washed in MilliQ water and digested through concentrated HNO_3_. After drying, samples were re-dissolved in 3 M HNO_3_ and processed through column chemistry (30 µl columns filled with Eichrom Sr-spec resin) for Sr separation^81^. The ^87^Sr/^86^Sr ratios were determined using a Neptune MC-ICPMS, housed at the Centro Interdipartimentale Grandi Strumenti of the University of Modena and Reggio Emilia^52,81,82^. Background and interference correction follow previous works^81,82^. Mass bias corrections used an exponential law and a ^88^Sr/^86^Sr ratio of 8.375209^12^. Repeated measures of NBS987 yielded an ^87^Sr/^86^Sr value of 0.710243 ± 0.000018 (2 SD; n = 8). All values were normalized to an NBS987 accepted value of 0.710248. Total laboratory Sr blanks did not exceed 200 pg.

## Supplementary Information

Supplementary Information 1.

Supplementary Information 2.
