# Supplementary material for: Tracing the mobility of a Late Epigravettian (~ 13 ka) male infant from Grotte di Pradis (Northeastern Italian Prealps) at high-temporal resolution
Source: Sci Rep. 2022 May 16;12:8104. doi: 10.1038/s41598-022-12193-6 (PMC9110381; doi:10.1038/s41598-022-12193-6)
Supplement: Supplementary file 2 — Supplementary Information 2. [file 41598_2022_12193_MOESM2_ESM.pdf]

Supplementary information for:

Tracing the mobility of a Late Epigravettian (~13 ka) male infant from Grotte di Pradis (Northeastern Italian Prealps) at high-temporal resolution

Federico Lugli, Alessia Nava, Rita Sorrentino, Antonino Vazzana, Eugenio Bortolini, Gregorio Oxilia, Silvestrini Sara, Nicola Nannini, Luca Bondioli, Helen Fewlass, Sahra Talamo, Edouard Bard, Lucia Mancini, Wolfgang Müller, Matteo Romandini, Stefano Benazzi

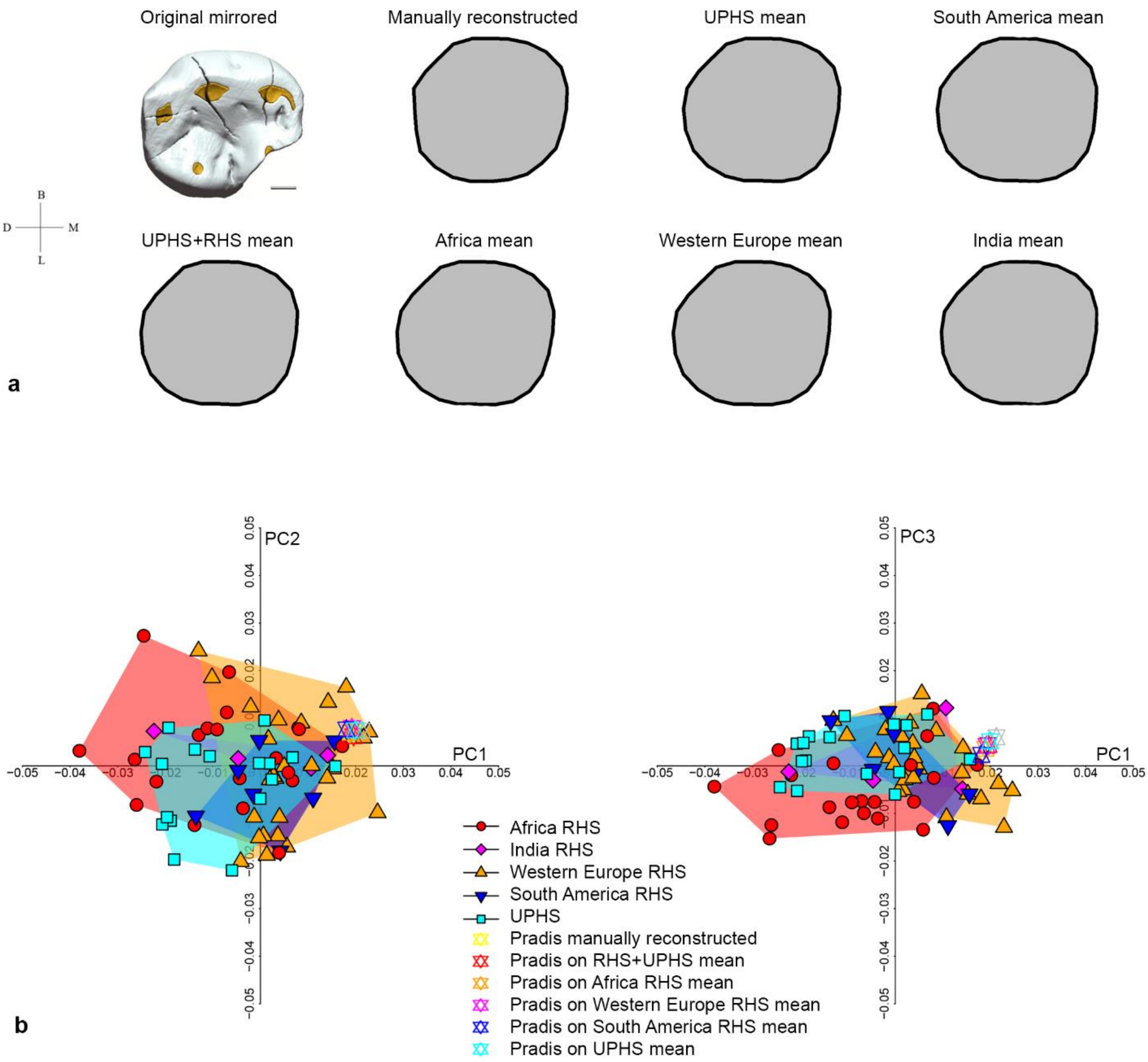

Supplementary Fig. S1. Pradis dm2 mirrored, manually reconstructed, and geometric morfometric reconstructed based on Upper Paleolithic *H. sapiens* (UPHS) mean, African recent *H. sapiens* (RHS) mean, Indian RHS mean, Western European RHS mean, South American RHS mean, and RHS - UPHS pooled groups (a). Principal component analysis (PCA) plot of PC1 vs. PC2 (on the left) and PC1 vs. PC3 (on the right) of the dm2 comparative sample in which Pradis reconstructions are projected. The stars represent Pradis right dm2 mirrored and reconstructed based on UPHS mean, African RHS mean, Indian RHS mean, Western European RHS mean, South American RHS mean, and RHS - UPHS pooled groups (b).

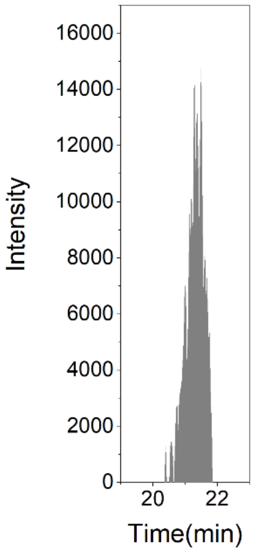

**RT (min)**	21.3
**RT (sec)**	1277.3
**Start RT**	20.7
**End RT**	21.8
**Base Peak**	396.7
**Peak Area**	485727.6
**Peak Height**	14940.9
**Baseline Width**	1.2
**SignalToNoise**	83.8
**m/z**	396.7073
**Peptide**	$M_{(ox)}$IRPPY

Supplementary Figure S2. LC-MS ion chromatograms of peptide M(ox)IRRPY (human AMELY) at m/z 396.7073 ($[M + 2\,H]^{+2}$).

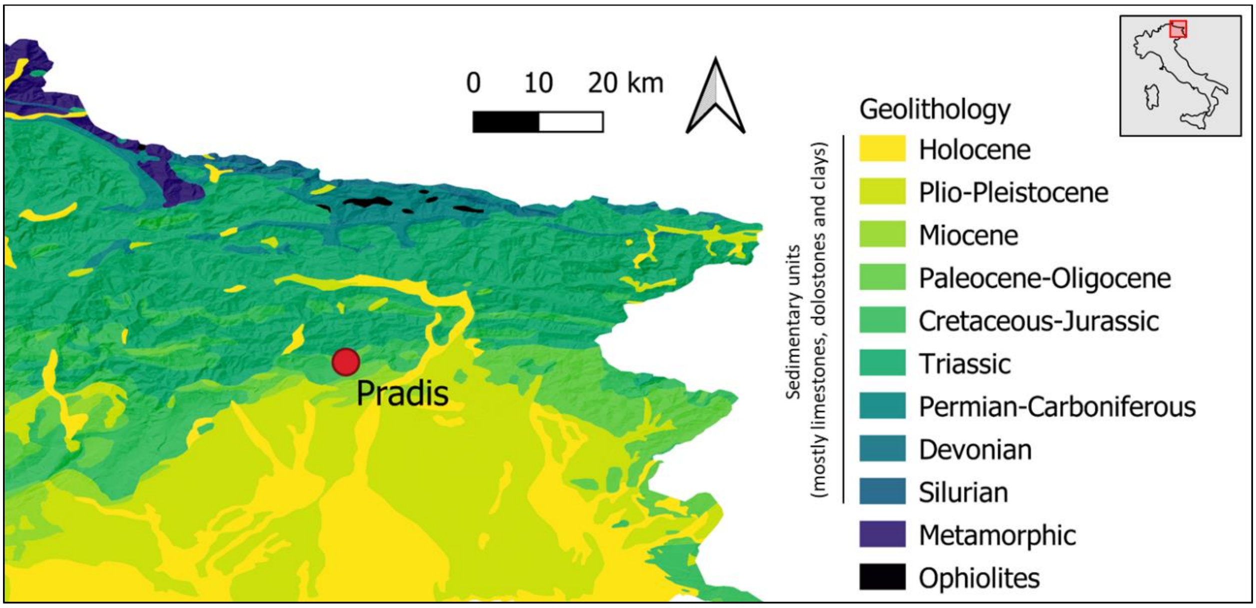

Supplementary Fig. S3. Geological map of Northeastern Italy, built in QGIS 3.18. This map is based on the geolithological map of Italy available at the Geoportale Nazionale (http://wms.pcn.minambiente.it/ogc?map=/ms_ogc/wfs/Carta_geolitologica.map).

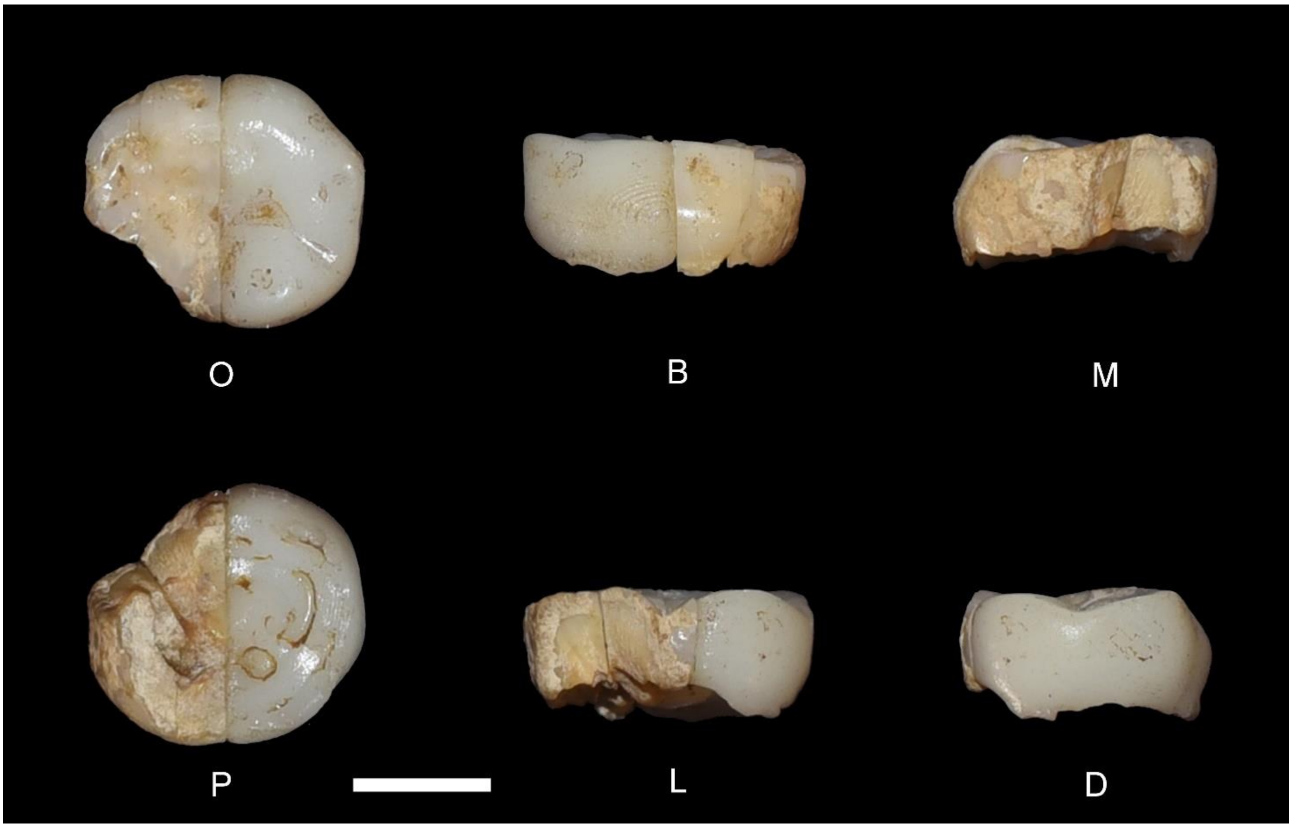

Supplementary Fig. S4. Photographic record of Pradis 1 tooth after the restoration protocol; scale bar is 5 mm; O = occlusal; B = buccal; M = mesial; L = lingual; D = distal; P = periapical.

Supplementary Table S1. Information on identified peptides within the chromatogram, using ICIS algorithm of Xcalibur.

RT (min)	RT (sec)	Start RT	End RT	Base Peak	Peak Area	Peak Height	Baseline Width	SignalToNoise	Peptide	m/z
21.8	1305.5	21.4	22.2	440.2	918747.1	33389.2	0.8	73.1	$SM_{(ox)}IRPPY$	440.2233
30.9	1856.5	30.3	31.3	540.3	1055280.0	37411.1	1.1	64.3	SIRPPYPSY	540.2796

Supplementary Table S2. Pretreatment and ^{14}C dating information for the Pradis 1 tooth. The ^{14}C dates were calibrated in OxCal v4.4 against the IntCal20 dataset (Bronk Ramsey, 2009; Reimer et al 2020).

Sample ID	Tooth sampled (mg)	Collagen yield (%)	C%	N%	C:N	$\delta^{13}C$ (‰)	$\delta^{15}N$ (‰)	^{14}C method	AMS lab number	^{14}C age (BP)	Error (y)	68.3% Calibrated range (cal BP)	95.4% Calibrated range (cal BP)
**Pradis 1**	102.2	5.9	39.2	14.6	3.1	-18.5	11.7	Graphite	MAMS-38332	11046	32		
								CO_2	AIX-12033	11217	124		
								Weighted mean (R_Combine)		11057	31	13068-12929	13088-12897
